# Supplementary material for: Architect: A tool for aiding the reconstruction of high-quality metabolic models through improved enzyme annotation
Source: PLoS Comput Biol. 2022 Sep 8;18(9):e1010452. doi: 10.1371/journal.pcbi.1010452 (PMC9488769; doi:10.1371/journal.pcbi.1010452)
Supplement: S1 Table — (DOCX) [file pcbi.1010452.s021.docx]

Supplemental Table 1: Breakdown of annotations of SwissProt sequences by individual and ensemble methods into true positives, true negatives, false positives and false negatives

|  |  | Enzymatic test set | | | Non-enzymatic test set | | |  |
| --- | --- | --- | --- | --- | --- | --- | --- | --- |
|  |  | # TPs | # FPs | # FNs | # prots annotated  as enzymes | # FP annotations | # TN proteins | Sum of FP annotations |
| Individual tools | CatFam | 31,364 | 3,644 | 13,209 | 5,090 | 5,159 | 288,977 | 8,803 |
|  | DETECT_all | 34,525 | 19,762 | 10,048 | 16,262 | 24,544 | 277,805 | 44,306 |
|  | DETECT_high | 34,158 | 1,476 | 10,415 | 2,110 | 2,163 | 291,957 | 3,639 |
|  | EFICAz_all | 32,466 | 5,099 | 12,107 | 3,909 | 4,283 | 290,158 | 9,382 |
|  | EFICAz_high | 31,606 | 2,569 | 12,967 | 1,497 | 1,505 | 292,570 | 4,074 |
|  | EnzDP_all | 39,649 | 134,150 | 4,924 | 49,229 | 151,923 | 244,838 | 286,073 |
|  | EnzDP_high | 37,930 | 821 | 6,643 | 1,587 | 1,760 | 292,480 | 2,581 |
|  | PRIAM_all | 43,099 | 2,430 | 1,474 | 4,293 | 4,870 | 289,774 | 7,300 |
|  | PRIAM_high | 42,845 | 1,227 | 1,728 | 2,149 | 2,597 | 291,918 | 3,824 |
| Ensemble methods | Majority rule | 42,030 | 1,362 | 2,543 | 7,763 | 8,501 | 286,304 | 9,863 |
|  | EC-specific tool | 43,430 | 479 | 1,143 | 10,500 | 10,961 | 283,567 | 11,440 |
|  | Naïve Bayes | 43,404 | 281 | 1,169 | 2,308 | 2,418 | 291,759 | 2,699 |
|  | L1-regression | 43,559 | 291 | 1,014 | 8,983 | 9,248 | 285,084 | 9,539 |
|  | L2-regression | 43,567 | 290 | 1,006 | 8,815 | 9,084 | 285,252 | 9,374 |
|  | Random forest | 43,595 | 297 | 978 | 8,601 | 8,899 | 285,466 | 9,196 |
