# Supplementary material for: Architect: A tool for aiding the reconstruction of high-quality metabolic models through improved enzyme annotation
Source: PLoS Comput Biol. 2022 Sep 8;18(9):e1010452. doi: 10.1371/journal.pcbi.1010452 (PMC9488769; doi:10.1371/journal.pcbi.1010452)
Supplement: S2 Table — (DOCX) [file pcbi.1010452.s022.docx]

Supplemental Table 2: Breakdown of organism-specific annotations by ensemble and individual tool into true positive, false positive and false negative. This comparison is done against UniProt annotations for sequences used in model reconstruction, and the predictions from the ensemble method come the naïve Bayes classifier for ECs in Architect’s training database, and PRIAM (high-confidence) otherwise.

|  | Tool | # TP | # FP in all | # FP on enzymes | # FP on non-enzymes  (# proteins in brackets) | # FP on proteins with partial ECs | # FN |
| --- | --- | --- | --- | --- | --- | --- | --- |
| *C. elegans* | DETECT | 825 | 753 | 113 | 495 (486) | 145 | 763 |
|  | EnzDP | 995 | 545 | 83 | 390 (335) | 72 | 593 |
|  | PRIAM | 1,170 | 667 | 194 | 413 (374) | 60 | 418 |
|  | Architect | 1,260 | 867 | 117 | 648 (613) | 102 | 328 |
| *N. meningitidis* | DETECT | 355 | 109 | 45 | 44 (43) | 20 | 206 |
|  | EnzDP | 415 | 75 | 28 | 28 (26) | 19 | 146 |
|  | PRIAM | 460 | 96 | 41 | 32 (29) | 23 | 101 |
|  | Architect | 482 | 108 | 42 | 44 (41) | 22 | 79 |
| *E. coli* | DETECT | 648 | 231 | 144 | 29 (28) | 58 | 762 |
|  | EnzDP | 1,087 | 186 | 120 | 33 (26) | 33 | 323 |
|  | PRIAM | 1,253 | 145 | 92 | 16 (15) | 37 | 157 |
|  | Architect | 1,257 | 173 | 102 | 24 (23) | 47 | 153 |
