# Supplementary material for: Architect: A tool for aiding the reconstruction of high-quality metabolic models through improved enzyme annotation
Source: PLoS Comput Biol. 2022 Sep 8;18(9):e1010452. doi: 10.1371/journal.pcbi.1010452 (PMC9488769; doi:10.1371/journal.pcbi.1010452)
Supplement: S3 Table — (DOCX) [file pcbi.1010452.s023.docx]

Supplemental Table 3: Comparisons of various aspects of model reconstruction for *C. elegans*, *N. meningitidis* and *E. coli.* The number of reactions in reconstructed models that are not blocked (and corresponding number of metabolites) is indicated within brackets, except in the case of automatically reconstructed *C. elegans* models (*). The number of exchange reactions added by Architect for deadend metabolites is given within brackets (**).

|  |  | Organism | | |
| --- | --- | --- | --- | --- |
|  |  | *C. elegans* | *N. meningitidis* | *E. coli* |
| Architect, CarveMe and PRIAM-based reconstructions | Source of protein sequences | WormBase database | Ensembl database | UniProt Proteome ID: UP000000625 |
|  | Num of protein sequences | 20,483 | 2,063 | 4,391 |
| Architect reconstruction | Biomass used | Main CarveMe biomass  (using KEGG identifiers when using KEGG database; same as for other organisms) | Gram-negative CarveMe biomass | Gram-negative CarveMe biomass |
|  | Media specified | None | Minimal media and aerobic conditions (M9) | Minimal media and anaerobic conditions (M9[-O2]); modified when simulating aerobic conditions |
|  | Penalty for addition of exchange reactions for deadend metabolites | 1 | 10 | 10 |
| Architect reconstruction using KEGG | Num of protein sequences | 1,125 | 372 | 915 |
|  | Num of reactions* | 1,432 | 900 (430) | 1,674 (869) |
|  | Num of metabolites* | 1,530 | 1,087 (378) | 1,692 (608) |
|  | Num of gap-filling reactions** | 37 (22) | 30 (2) | 8 (2) |
| Architect reconstruction using KEGG and predictions from individual tools | Num of reactions* | DETECT: 1,023 EnzDP: 1,272 PRIAM: 1,355 | DETECT: 812 (410) EnzDP: 839 (405) PRIAM: 880 (426) | DETECT: 1,135 (525) EnzDP: 1,510 (738) PRIAM: 1,701 (882) |
|  | Num of metabolites | DETECT: 1,220 EnzDP: 1,402 PRIAM: 1,474 | DETECT: 978 (363) EnzDP: 1,026 (359) PRIAM: 1,066 (376) | DETECT: 1,342 (422) EnzDP: 1,593 (531) PRIAM: 1,724 (622) |
|  | Num of gap-filling reactions** | DETECT: 44 (26) EnzDP: 43 (26) PRIAM: 40 (24) | DETECT: 69 (2)  EnzDP: 44 (2) PRIAM: 35 (2) | DETECT: 57 (2) EnzDP: 22 (2) PRIAM: 12 (2) |
| Architect reconstruction using BiGG | Num of protein sequences | 736 | 472 | 1,475 |
|  | Num of reactions* | 1,407 | 1,622 (568) | 3,032 (1,688) |
|  | Num of metabolites* | 1,312 | 1,518 (444) | 2,049 (1,014) |
|  | Num of gap-filling reactions** | 24 (7) | 49 (0) | 14 (0) |
| Architect reconstruction using BiGG and predictions from individual tools | Num of reactions* | DETECT: 1,327 EnzDP: 1,367 PRIAM: 1,372 | DETECT: 1,553 (549) EnzDP: 1,573 (548) PRIAM: 1,587 (562) | DETECT: 2,697 (1266) EnzDP: 2,938 (1,556) PRIAM: 3,025 (1,714) |
|  | Num of metabolites | DETECT: 1,284 EnzDP: 1,303 PRIAM: 1,301 | DETECT: 1,486 (437) EnzDP: 1,490 (429) PRIAM: 1,506 (442) | DETECT: 1,995 (828) EnzDP: 2,041 (967) PRIAM: 2,050 (1,021) |
|  | Num of gap-filling reactions** | DETECT: 36 (10) EnzDP: 32 (13) PRIAM: 27 (10) | DETECT: 79 (0) EnzDP: 64 (0) PRIAM: 53 (0) | DETECT: 56 (0) EnzDP: 22 (0) PRIAM: 14 (0) |
| CarveMe reconstruction | Biomass used | Main CarveMe biomass | Gram-negative biomass | Gram-negative biomass |
|  | Num of genes/protein sequences | 562 | 613 | 1,639 |
|  | Num of reactions* | 1,538 | 4,345 (3,256) | 4,345 (3,256) |
|  | Num of metabolites* | 1,100 | 2,383 (1,767) | 2,383 (1,767) |
| ModelSEED reconstruction | Biomass used | Generic biomass | Gram-negative biomass | Gram-negative biomass |
|  | Num of genes/protein sequences | 818 | 509 | 930 |
|  | Num of reactions* | 1,115 | 1,084 (587) | 1,647 (972) |
|  | Num of metabolites* | 1,238 | 1,253 (558) | 1,698 (804) |
| Reconstruction using PRIAM | Num of protein sequences | 920 | 452 | 1,052 |
|  | Num of reactions | 1,387 | 867 | 1,890 |
|  | Num of metabolites | 1,397 | 935 | 1,717 |
| Gold-standard models | Provenance | WormJam [1]; version 2019_01_01 from *https://gh.wormjam.life* | Nmb_iTM560 [2] | iML1515 [3] |
|  | Num of genes | 1,520 | 559 | 1,515 |
|  | Num of reactions* | 3,632 (2,947) | 1,527 (not available) | 2,719 (2,459) |
|  | Num of metabolites* | 2,833 | 1,297 | 1,192 |
| UniProt gold-standard annotations | Provenance | Uniprot | Uniprot | Using annotations for UP000000625 in SwissProt |
|  | Num of protein sequences with EC annotations when comparing against KEGG-based Architect | 1,446 | 495 | 1,123 |
|  | Num of protein sequences with EC annotations when comparing against BiGG-based Architect | 659 | 504 | 1,111 |
| Essentiality results | Provenance | Not applicable | [2] | [3] |

Bibliography

1. Witting, M., et al., *Modeling Meets Metabolomics-The WormJam Consensus Model as Basis for Metabolic Studies in the Model Organism Caenorhabditis elegans.* Front Mol Biosci, 2018. **5**: p. 96.

2. Mendum, T.A., et al., *Interrogation of global mutagenesis data with a genome scale model of Neisseria meningitidis.* Genome Biology, 2011. **12**.

3. Monk, J.M., et al., *iML1515, a knowledgebase that computes Escherichia coli traits.* Nat Biotechnol, 2017. **35**(10): p. 904-908.
