# Supplementary material for: Architect: A tool for aiding the reconstruction of high-quality metabolic models through improved enzyme annotation
Source: PLoS Comput Biol. 2022 Sep 8;18(9):e1010452. doi: 10.1371/journal.pcbi.1010452 (PMC9488769; doi:10.1371/journal.pcbi.1010452)
Supplement: S4 Table — (DOCX) [file pcbi.1010452.s024.docx]

Supplemental Table 4: Summary of MEMOTE [1] results for models reconstructed using Architect (KEGG and BiGG universes), CarveMe, PRIAM and ModelSEED and those manually reconstructed. MEMOTE tests were preferably run on command line using v0.13.0 of the software and a command of the form memote report snapshot --filename output.html model.xml. However, in the case of timeout or out-of-memory error, reports were obtained from https://memote.io/ (also v0.13.0). Before the inclusion of Systems Biology Ontology terms to each gene, reaction and species element (SBO: 0000243, SBO: 0000375 and SBO: 0000247 respectively), Architect models scored 0% on gene and SBO annotation. We note that MEMOTE computes the number of blocked reactions using flux variability analysis [2] with all model boundaries open, whereas our identification of blocked reactions (Supplementary Table 2) does not involve prior modification of model boundaries.

|  |  | Method of reconstruction | | | | | |
| --- | --- | --- | --- | --- | --- | --- | --- |
|  |  | Architect-KEGG | Architect-BiGG | CarveMe | PRIAM | ModelSEED | Manual |
| *C. elegans* | Fraction of reactions without GPR rules | 14% | 5% | 27% | 6% | 7% | 14% |
|  | Fraction of blocked reactions | 55% | 65% | 3% | 43% | 39% | 27% |
|  | Consistency | 31% | 38% | 32% | 14% | 82% | 38% |
|  | Metabolite annotation | 48% | 73% | 25% | 25% | 25% | 54% |
|  | Reaction annotation | 76% | 73% | 25% | 25% | 27% | 39% |
|  | Gene annotation | 33% | 33% | 0% | 0% | 0% | 33% |
|  | SBO annotation | 45% | 45% | 0% | 0% | 10% | 0% |
|  | Overall score | 42% | 46% | 16% | 9% | 40% | 23% |
| *N. meningitidis* | Fraction of reactions without GPR rules | 23% | 6% | 62% | 9% | 5% | 16% |
|  | Fraction of blocked reactions | 52% | 60% | 0%* | 53% | 43% | 50% |
|  | Consistency | 81% | 41% | 36% | 15% | 82% | 42% |
|  | Metabolite annotation | 48% | 73% | 25% | 25% | 25% | 25% |
|  | Reaction annotation | 76% | 73% | 25% | 25% | 26% | 29% |
|  | Gene annotation | 33% | 33% | 0% | 0% | 0% | 0% |
|  | SBO annotation | 45% | 45% | 0% | 0% | 10% | 10% |
|  | Overall score | 61% | 47% | 18% | 10% | 40% | 25% |
| *E. coli* | Fraction of reactions without GPR rules | 12% | 2% | 33% | 7% | 7% | 4% |
|  | Fraction of blocked reactions | 48% | 21% | 0%* | 38% | 37% | 10% |
|  | Consistency | 77% | 37% | 27% | 15% | 78% | 98% |
|  | Metabolite annotation | 48% | 73% | 25% | 25% | 25% | 79% |
|  | Reaction annotation | 77% | 74% | 25% | 25% | 27% | 76% |
|  | Gene annotation | 33% | 33% | 0% | 0% | 0% | 67% |
|  | SBO annotation | 45% | 45% | 0% | 0% | 10% | 36% |
|  | Overall score | 60% | 46% | 14% | 10% | 38% | 68% |

Bibliography

1. Lieven, C., et al., *MEMOTE for standardized genome-scale metabolic model testing.* Nat Biotechnol, 2020. **38**(3): p. 272-276.

2. Mahadevan, R. and C.H. Schilling, *The effects of alternate optimal solutions in constraint-based genome-scale metabolic models.* Metab Eng, 2003. **5**(4): p. 264-76.
