# Supplementary material for: Architect: A tool for aiding the reconstruction of high-quality metabolic models through improved enzyme annotation
Source: PLoS Comput Biol. 2022 Sep 8;18(9):e1010452. doi: 10.1371/journal.pcbi.1010452 (PMC9488769; doi:10.1371/journal.pcbi.1010452)
Supplement: S5 Table — (DOCX) [file pcbi.1010452.s025.docx]

Supplemental Table 5: Overlap of *in silico* determined essential genes with those found essential *in vivo*.

|  | Method | # TP | # FP | # FN  (altogether) | # essential genes not included  in output model | Precision | Recall | Recall (only considering genes included in model) |
| --- | --- | --- | --- | --- | --- | --- | --- | --- |
| *N. meningitidis* | Architect-KEGG | 119 | 35 | 121 | 54 | 77.3% | 49.6% | 64.0% |
|  | Architect-BiGG | 132 | 34 | 108 | 68 | 79.5% | 55% | 76.7% |
|  | CarveMe | 83 | 13 | 157 | 20 | 86.5% | 34.6% | 37.7% |
|  | ModelSEED | 130 | 40 | 110 | 62 | 76.5% | 54.2% | 73.0% |
|  | Arch-DETECT (KEGG) | 86 | 24 | 154 | 80 | 78.2% | 35.8% | 53.8% |
|  | Arch-EnzDP (KEGG) | 102 | 31 | 138 | 77 | 76.7% | 42.5% | 62.6% |
|  | Arch-PRIAM (KEGG) | 109 | 34 | 131 | 67 | 76.2% | 45.4% | 63.0% |
|  | Arch-DETECT (BiGG) | 110 | 27 | 130 | 88 | 80.3% | 45.8% | 72.4% |
|  | Arch-EnzDP (BiGG) | 119 | 28 | 121 | 88 | 81.0% | 49.6% | 78.3% |
|  | Arch-PRIAM (BiGG) | 124 | 35 | 116 | 77 | 78.0% | 51.7% | 76.1% |
| *E. coli* | Architect-KEGG | 86 | 15 | 163 | 50 | 85.1% | 34.5% | 43.2% |
|  | Architect-BiGG | 119 | 22 | 130 | 58 | 84.4% | 47.8% | 62.3% |
|  | CarveMe | 74 | 6 | 175 | 39 | 92.5% | 29.7% | 35.2% |
|  | ModelSEED | 110 | 52 | 139 | 66 | 67.9% | 44.2% | 60.1% |
|  | Arch-DETECT (KEGG) | 69 | 13 | 180 | 86 | 84.1% | 27.7% | 42.3% |
|  | Arch-EnzDP (KEGG) | 84 | 14 | 165 | 65 | 85.7% | 33.7% | 45.7% |
|  | Arch-PRIAM (KEGG) | 82 | 20 | 167 | 54 | 80.4% | 32.9% | 42.1% |
|  | Arch-DETECT (BiGG) | 101 | 20 | 148 | 88 | 83.5% | 40.6% | 62.7% |
|  | Arch-EnzDP (BiGG) | 113 | 28 | 136 | 71 | 80.1% | 45.4% | 63.5% |
|  | Arch-PRIAM (BiGG) | 121 | 22 | 128 | 60 | 84.6% | 48.6% | 64.0% |

Note: Calculations of the percentage of genes experimentally found to be essential and incorporated into the Architect models (as listed in the main manuscript) were calculated as: 1- (number of essential genes not included in model / number of all essential genes). The number of all essential genes was defined as the sum of true positives and false negatives (altogether).

Calculations of the percentage of genes reported in the main manuscript not predicted to be essential were calculated as: the number of false negatives (altogether) / the number of (experimentally identified) essential genes included in the model. The number of (experimentally identified) essential genes in the model was obtained as the sum of true positives and false negatives minus the number of essential genes not included in the model.
