# Supplementary material for: Architect: A tool for aiding the reconstruction of high-quality metabolic models through improved enzyme annotation
Source: PLoS Comput Biol. 2022 Sep 8;18(9):e1010452. doi: 10.1371/journal.pcbi.1010452 (PMC9488769; doi:10.1371/journal.pcbi.1010452)
Supplement: S6 Table — (DOCX) [file pcbi.1010452.s026.docx]

Supplemental Table 6: Details of *E. coli* phenotypes tested *in silico*. The ability of Architect, CarveMe and ModelSEED model to grow on the following individual compounds was tested. This Biolog data was obtained from [1].

| Array type | Sustains growth? | Compound name | BiGG | ModelSEED | KEGG |
| --- | --- | --- | --- | --- | --- |
| Carbon | No | γ-Amino ButyricAcid | 4abut_e | cpd00281 | C00334 |
| Carbon | Yes | Adenosine | adn_e | cpd00182 | C00212 |
| Carbon | Yes | α-Keto-GlutaricAcid | akg_e | cpd00024 | C00026 |
| Carbon | Yes | D-Alanine | ala__D_e | cpd00117 | C00133 |
| Carbon | No | Citric Acid | cit_e | cpd00137 | C00158 |
| Carbon | No | 2-Aminoethanol | etha_e | cpd00162 | C00189 |
| Carbon | Yes | Fumaric Acid | fum_e | cpd00106 | C00122 |
| Carbon | Yes | D-GalactonicAcid-γ-Lactone | galctn__D_e | cpd00653 | C00880 |
| Carbon | No | Dulcitol | galt_e | cpd01171 | C01697 |
| Carbon | Yes | D-GalacturonicAcid | galur_e | cpd00280 | C00333 |
| Carbon | Yes | D-Gluconic Acid | glcn_e | cpd00222 | C00257 |
| Carbon | Yes | D-GlucuronicAcid | glcur_e | cpd00164 | C00191 |
| Carbon | Yes | Glycine | gly_e | cpd00033 | C00037 |
| Carbon | No | Glycerol | glyc_e | cpd00100 | C00116 |
| Carbon | No | D,L-α-Glycerol-Phosphate | glyc3p_e | cpd00080 | C00093 |
| Carbon | Yes | Inosine | ins_e | cpd00246 | C00294 |
| Carbon | Yes | L-Lactic Acid | lac__L_e | cpd00159 | C00186 |
| Carbon | Yes | L-Malic Acid | mal__L_e | cpd00130 | C00149 |
| Carbon | Yes | D-Melibiose | melib_e | cpd03198 | C05402 |
| Carbon | Yes | D-Mannitol | mnl_e | cpd00314 | C00392 |
| Carbon | Yes | D-Sorbitol | sbt__D_e | cpd00588 | C00794 |
| Carbon | Yes | D-Serine | ser__D_e | cpd00550 | C00740 |
| Carbon | Yes | Succinic Acid | succ_e | cpd00036 | C00042 |
| Carbon | No | Sucrose | sucr_e | cpd00076 | C00089 |
| Carbon | No | L-Threonine | thr__L_e | cpd00161 | C00188 |
| Carbon | Yes | Thymidine | thymd_e | cpd00184 | C00214 |
| Carbon | Yes | D-Trehalose | tre_e | cpd00794 | C01083 |
| Carbon | Yes | Uridine | uri_e | cpd00249 | C00299 |
| Carbon | Yes | D-Xylose | xyl__D_e | cpd00154 | C00181 |
| Carbon | Yes | N-Acetyl-DGlucosamine | acgam_e | cpd00122 | C00140 |
| Carbon | Yes | L-Alanine | ala__L_e | cpd00035 | C00041 |
| Carbon | No | L-Arginine | arg__L_e | cpd00051 | C00062 |
| Carbon | No | L-Asparagine | asn__L_e | cpd00132 | C00152 |
| Carbon | Yes | L-Aspartic Acid | asp__L_e | cpd00041 | C00049 |
| Carbon | Yes | D-Fructose | fru_e | cpd00082 | C00095 |
| Carbon | Yes | Glucose-6-Phosphate | g6p_e | cpd00079 | C00092 |
| Carbon | Yes | D-Galactose | gal_e | cpd00108 | C00124 |
| Carbon | Yes | α-D-Glucose | glc__D_e | cpd00027 | C00031 |
| Carbon | Yes | L-Glutamine | gln__L_e | cpd00053 | C00064 |
| Carbon | No | L-Glutamic Acid | glu__L_e | cpd00023 | C00025 |
| Carbon | Yes | Glycolic Acid | glyclt_e | cpd00139 | C00160 |
| Carbon | No | L-Histidine | his__L_e | cpd00119 | C00135 |
| Carbon | Yes | α-D-Lactose | lcts_e | cpd00208 | C00243 |
| Carbon | No | L-Lysine | lys__L_e | cpd00039 | C00047 |
| Carbon | Yes | Maltose | malt_e | cpd00179 | C00208 |
| Carbon | Yes | D-Mannose | man_e | cpd00138 | C00159 |
| Carbon | No | L-Methionine | met__L_e | cpd00060 | C00073 |
| Carbon | No | L-Phenylalanine | phe__L_e | cpd00066 | C00079 |
| Carbon | No | L-Proline | pro__L_e | cpd00129 | C00148 |
| Carbon | No | Putrescine | ptrc_e | cpd00118 | C00134 |
| Carbon | Yes | L-Rhamnose | rmn_e | cpd00396 | C00507 |
| Carbon | Yes | L-Serine | ser__L_e | cpd00054 | C00065 |
| Nitrogen | No | Adenosine | adn_e | cpd00182 | C00212 |
| Nitrogen | Yes | D-Alanine | ala__D_e | cpd00117 | C00133 |
| Nitrogen | No | Ethanolamine | etha_e | cpd00162 | C00189 |
| Nitrogen | Yes | Glycine | gly_e | cpd00033 | C00037 |
| Nitrogen | No | Inosine | ins_e | cpd00246 | C00294 |
| Nitrogen | Yes | D-Serine | ser__D_e | cpd00550 | C00740 |
| Nitrogen | Yes | L-Threonine | thr__L_e | cpd00161 | C00188 |
| Nitrogen | No | Thymidine | thymd_e | cpd00184 | C00214 |
| Nitrogen | No | Uridine | uri_e | cpd00249 | C00299 |
| Nitrogen | Yes | Cytosine | csn_e | cpd00307 | C00380 |
| Nitrogen | Yes | Cytidine | cytd_e | cpd00367 | C00475 |
| Nitrogen | Yes | Guanosine | gsn_e | cpd00311 | C00387 |
| Nitrogen | No | Uracil | ura_e | cpd00092 | C00106 |
| Nitrogen | Yes | Xanthine | xan_e | cpd00309 | C00385 |
| Nitrogen | Yes | Xanthosine | xtsn_e | cpd01217 | C01762 |
| Nitrogen | Yes | N-Acetyl-D-Glucosamine | acgam_e | cpd00122 | C00140 |
| Nitrogen | Yes | L-Alanine | ala__L_e | cpd00035 | C00041 |
| Nitrogen | Yes | L-Arginine | arg__L_e | cpd00051 | C00062 |
| Nitrogen | Yes | L-Asparagine | asn__L_e | cpd00132 | C00152 |
| Nitrogen | Yes | L-AsparticAcid | asp__L_e | cpd00041 | C00049 |
| Nitrogen | Yes | L-Glutamine | gln__L_e | cpd00053 | C00064 |
| Nitrogen | Yes | L-GlutamicAcid | glu__L_e | cpd00023 | C00025 |
| Nitrogen | Yes | L-Histidine | his__L_e | cpd00119 | C00135 |
| Nitrogen | Yes | L-Lysine | lys__L_e | cpd00039 | C00047 |
| Nitrogen | Yes | L-Methionine | met__L_e | cpd00060 | C00073 |
| Nitrogen | Yes | L-Phenylalanine | phe__L_e | cpd00066 | C00079 |
| Nitrogen | Yes | L-Proline | pro__L_e | cpd00129 | C00148 |
| Nitrogen | Yes | Putrescine | ptrc_e | cpd00118 | C00134 |
| Nitrogen | Yes | L-Serine | ser__L_e | cpd00054 | C00065 |
| Nitrogen | Yes | L-Tryptophan | trp__L_e | cpd00065 | C00078 |
| Nitrogen | Yes | L-Tyrosine | tyr__L_e | cpd00069 | C00082 |
| Sulphur | Yes | Thiosulfate | tsul_e | cpd00268 | C00320 |
| Sulphur | Yes | L-Methionine | met__L_e | cpd00060 | C00073 |
| Sulphur | Yes | L-MethionineSulfoxide | metsox_S__L_e | cpd01914 | C02989 |
| Sulphur | Yes | Sulfate | so4_e | cpd00048 | C00059 |
| Phosphorus | Yes | D-Mannose-6-Phosphate | man6p_e | cpd00235 | C00275 |
| Phosphorus | Yes | D-Glucose-6-Phosphate | g6p_e | cpd00079 | C00092 |
| Phosphorus | Yes | Phosphate | pi_e | cpd00009 | C00009 |

Bibliography

1. Feist, A.M., et al., *A genome-scale metabolic reconstruction for Escherichia coli K-12 MG1655 that accounts for 1260 ORFs and thermodynamic information.* Mol Syst Biol, 2007. **3**: p. 121.
