# Supplementary material for: Architect: A tool for aiding the reconstruction of high-quality metabolic models through improved enzyme annotation
Source: PLoS Comput Biol. 2022 Sep 8;18(9):e1010452. doi: 10.1371/journal.pcbi.1010452 (PMC9488769; doi:10.1371/journal.pcbi.1010452)
Supplement: S2 Data — The reports of the manually curated models are found in the folder named Benchmarks, and those reports detailing statistics of the automatically generated models are located in the folders named according to the tool utilized. MEMOTE v0.13.0 was used to generate the reports. (ZIP) [file pcbi.1010452.s028.zip › Architect_BiGG/Nmeningitidis.html]

MemoteReportApp

 Architect\_model\_0  Expand AllReadme2022-06-24 17:20

Independent Section  Contains tests that are independent of the class of modeled organism, a model's complexity or types of identifiers that are used to describe its components. Parameterization or initialization of the network is not required. See readme for more details. 

## Consistency

Stoichiometric Consistency |

0.0% |

X3

Mass Balance |

0.0% |

Charge Balance |

100.0% |

Metabolite Connectivity |

100.0% |

Unbounded Flux In Default Medium |

86.5% |

---

Sub Total |

41% |

X3

The Sub Total is the result of the following calculation. For more information please click on "Readme" in the top left of the report.

86.51+(2⋅100)+(2⋅0)(5⋅100)=40.9386.51+(2⋅100)+(2⋅0)(5⋅100)=40.93

## Annotation - Metabolites

Presence of Metabolite Annotation |

100.0% |

Metabolite Annotations Per Database | Info |

 pubchem.compound |

0.0% |

 kegg.compound |

65.3% |

 seed.compound |

75.3% |

 inchikey |

0.0% |

 inchi |

0.0% |

 chebi |

71.5% |

 hmdb |

53.2% |

 reactome |

37.4% |

 metanetx.chemical |

99.9% |

 bigg.metabolite |

5.7% |

 biocyc |

0.0% |

Metabolite Annotation Conformity Per Database | Info |

 pubchem.compound |

0.0% |

 kegg.compound |

100.0% |

 seed.compound |

100.0% |

 inchikey |

0.0% |

 inchi |

0.0% |

 chebi |

100.0% |

 hmdb |

0.0% |

 reactome |

100.0% |

 metanetx.chemical |

99.8% |

 bigg.metabolite |

100.0% |

 biocyc |

0.0% |

Uniform Metabolite Identifier Namespace |

100.0% |

---

Sub Total |

73% |

The Sub Total is the result of the following calculation. For more information please click on "Readme" in the top left of the report.

65.35+75.30+71.48+53.23+37.35+99.93+5.73+99.80+(7⋅100)+(9⋅0)(24⋅100)=72.9165.35+75.30+71.48+53.23+37.35+99.93+5.73+99.80+(7⋅100)+(9⋅0)(24⋅100)=72.91

## Annotation - Reactions

Presence of Reaction Annotation |

100.0% |

Reaction Annotations Per Database | Info |

 rhea |

26.6% |

 kegg.reaction |

21.6% |

 seed.reaction |

38.9% |

 metanetx.reaction |

66.3% |

 bigg.reaction |

36.1% |

 reactome |

0.0% |

 ec-code |

30.6% |

 brenda |

0.0% |

 biocyc |

0.0% |

Reaction Annotation Conformity Per Database | Info |

 rhea |

100.0% |

 kegg.reaction |

100.0% |

 seed.reaction |

100.0% |

 metanetx.reaction |

100.0% |

 bigg.reaction |

100.0% |

 reactome |

0.0% |

 ec-code |

100.0% |

 brenda |

0.0% |

 biocyc |

0.0% |

Uniform Reaction Identifier Namespace |

100.0% |

---

Sub Total |

73% |

The Sub Total is the result of the following calculation. For more information please click on "Readme" in the top left of the report.

26.63+21.58+38.90+66.34+36.07+30.58+(8⋅100)+(6⋅0)(20⋅100)=72.7826.63+21.58+38.90+66.34+36.07+30.58+(8⋅100)+(6⋅0)(20⋅100)=72.78

## Annotation - Genes

Presence of Gene Annotation |

100.0% |

Gene Annotations Per Database | Info |

 refseq |

0.0% |

 uniprot |

0.0% |

 ecogene |

0.0% |

 kegg.genes |

0.0% |

 ncbigi |

0.0% |

 ncbigene |

0.0% |

 ncbiprotein |

0.0% |

 ccds |

0.0% |

 hprd |

0.0% |

 asap |

0.0% |

Gene Annotation Conformity Per Database | Info |

 refseq |

0.0% |

 uniprot |

0.0% |

 ecogene |

0.0% |

 kegg.genes |

0.0% |

 ncbigi |

0.0% |

 ncbigene |

0.0% |

 ncbiprotein |

0.0% |

 ccds |

0.0% |

 hprd |

0.0% |

 asap |

0.0% |

---

Sub Total |

33% |

The Sub Total is the result of the following calculation. For more information please click on "Readme" in the top left of the report.

(1⋅100)+(20⋅0)(21⋅100)=33.33(1⋅100)+(20⋅0)(21⋅100)=33.33

## Annotation - SBO Terms

Metabolite General SBO Presence |

100.0% |

Metabolite SBO:0000247 Presence |

100.0% |

Reaction General SBO Presence |

100.0% |

Metabolic Reaction SBO:0000176 Presence |

0.0% |

Transport Reaction SBO:0000185 Presence |

Skipped |

Exchange Reaction SBO:0000627 Presence |

0.0% |

Demand Reaction SBO:0000628 Presence |

Skipped |

Sink Reactions SBO:0000632 Presence |

Skipped |

Gene General SBO Presence |

100.0% |

Gene SBO:0000243 Presence |

100.0% |

Biomass Reactions SBO:0000629 Presence |

Skipped |

---

Sub Total |

45% |

X2

The Sub Total is the result of the following calculation. For more information please click on "Readme" in the top left of the report.

(5⋅100)+(6⋅0)(11⋅100)=45.45(5⋅100)+(6⋅0)(11⋅100)=45.45

---

---

Total Score |

47% |

The Total Score is the result of the following calculation. For more information please click on "Readme" in the top left of the report.

(3⋅40.93)+(1⋅72.91)+(1⋅72.78)+(1⋅33.33)+(2⋅45.45)(3⋅100)+(1⋅100)+(1⋅100)+(1⋅100)+(2⋅100)=47.08(3⋅40.93)+(1⋅72.91)+(1⋅72.78)+(1⋅33.33)+(2⋅45.45)(3⋅100)+(1⋅100)+(1⋅100)+(1⋅100)+(2⋅100)=47.08

---

Total Score 

47%

Score per Category 

Export

0%20%40%60%80%100%scoreconsistencyannotation\_metannotation\_rxnannotation\_geneannotation\_sbosection

Specific Section  Covers general statistics and specific aspects of a metabolic network that are not universally applicable. See readme for more details. 

SBML

SBML Level and Version |

Errored |

FBC enabled |

Errored |

Basic Information

Model Identifier |

Architect\_model\_0 |

Total Metabolites |

1,518 |

Total Reactions |

1,622 |

Total Genes |

472 |

Total Compartments |

1 |

Metabolic Coverage |

3.44 |

Uncoserved Metabolites |

688 |

Minimal Inconsistent Net Stoichiometries |

Skipped |

Metabolite Information

Unique Metabolites |

1,518 |

Duplicate Metabolites in Identical Compartments |

0 |

Metabolites without Charge |

0 |

Metabolites without Formula |

0 |

Medium Components |

18 |

Reaction Information

Purely Metabolic Reactions |

1,126 |

Purely Metabolic Reactions with Constraints |

6 |

Transport Reactions |

0 |

Transport Reactions with Constraints |

0 |

Reactions With Partially Identical Annotations |

0.11 |

Duplicate Reactions |

0.00 |

Reactions With Identical Genes |

0.49 |

Gene-Protein-Reaction (GPR) Associations

Reactions without GPR |

96 |

Fraction of Transport Reactions without GPR |

1.00 |

Enzyme Complexes |

Errored |

Biomass

Biomass Reactions Identified |

0 |

Biomass Consistency |

Skipped |

Biomass Production In Default Medium |

Skipped |

Unrealistic Growth Rate In Default Medium |

Skipped |

Biomass Production In Complete Medium |

Skipped |

Blocked Biomass Precursors In Default Medium |

Skipped |

Blocked Biomass Precursors In Complete Medium |

Skipped |

Ratio of Direct Metabolites in Biomass Reaction |

Skipped |

Number of Missing Essential Biomass Precursors |

Skipped |

Energy Metabolism

Non-Growth Associated Maintenance Reaction |

Errored |

Growth-associated Maintenance in Biomass Reaction |

Skipped |

Number of Reversible Oxygen-Containing Reactions |

3 |

Erroneous Energy-generating Cycles | Info |

 MNXM3 |

Skipped |

 MNXM63 |

Skipped |

 MNXM51 |

Skipped |

 MNXM121 |

Skipped |

 MNXM423 |

Skipped |

 MNXM6 |

Skipped |

 MNXM10 |

Skipped |

 MNXM38 |

Skipped |

 MNXM208 |

Skipped |

 MNXM191 |

Skipped |

 MNXM223 |

Skipped |

 MNXM7517 |

Skipped |

 MNXM12233 |

Skipped |

 MNXM558 |

Skipped |

 MNXM21 |

Skipped |

 MNXM89557 |

Skipped |

Network Topology

Universally Blocked Reactions |

975 |

Orphan Metabolites |

162 |

Dead-end Metabolites |

192 |

Stoichiometrically Balanced Cycles |

70 |

Metabolite Production In Complete Medium |

492 |

Metabolite Consumption In Complete Medium |

581 |

Matrix Conditioning

Ratio Min/Max Non-Zero Coefficients |

0.00 |

Independent Conservation Relations |

128 |

Rank |

1390 |

Degrees Of Freedom |

232 |

Experimental Data Comparison

Growth Prediction |

Skipped |

Gene Essentiality Prediction |

Skipped |

Misc. Tests

Environment Python Version 3.8.12 Platform Linux Memote Version 0.13.0

Package Versions

{"memote":"0.13.0","click":"7.1.2","click-configfile":"0.2.3","click-log":"0.3.2","six":"1.16.0","future":"0.18.2","pytest":"6.2.5","gitpython":"3.1.24","pandas":"1.3.4","Jinja2":"3.0.2","cookiecutter":"1.7.3","cobra":"0.22.1","ruamel.yaml":"0.17.16","travis-encrypt":"1.1.2","sympy":"1.9","sqlalchemy":"1.4.26","requests":"2.26.0","importlib-resources":"5.3.0","numpydoc":"1.1.0","pylru":"1.2.0","goodtables":"2.5.4","depinfo":"1.7.0","black ; extra == 'development'":"not installed","isort":"5.9.3","tox ; extra == 'development'":"not installed","pip":"21.3.1","setuptools":"58.3.0","wheel":"0.37.0"}
