# Supplementary material for: Architect: A tool for aiding the reconstruction of high-quality metabolic models through improved enzyme annotation
Source: PLoS Comput Biol. 2022 Sep 8;18(9):e1010452. doi: 10.1371/journal.pcbi.1010452 (PMC9488769; doi:10.1371/journal.pcbi.1010452)
Supplement: Attachment — Submitted filename: Response_To_Reviewers.docx [file pcbi.1010452.s030.docx]

*Reviewer #1: Architect: a tool for producing high-quality metabolic models through improved enzyme annotation

Here the authors present “Architect”, a new automated tool for building genome-scale metabolic models. It differs from other tools by using an ensemble-based enzyme annotation strategy, applying multiple annotation algorithms and using a variety of strategies to combine the resulting annotations into a single ideal annotation selected by their algorithm. The approach displays extremely high recall and precision compared with other methods.*

***C1:*** *Overall, it’s clear the annotation approach proposed works very well, as least for the model genomes studied (and for swissprot proteins). Of course, EC alone is not always a perfectly precise description of function, as often many reactions will map to a single EC, and not all are always correct.*

**A1**: We thank the reviewer for their constructive comment. We acknowledge that the Enzyme Commission number is imperfect and does not necessarily give a precise description of function and have added text to the discussion to this effect (lines 461-466).

***C2:*** *The idea of using multiple annotation algorithms as an ensemble to arrive at a better model is not entirely novel and has been described before, and the literature was not cited in the present manuscript. See “Combining multiple functional annotation tools increases coverage of metabolic annotation” as an example.*

**A2**: We thank the reviewer for pointing out relevant literature regarding combining predictions from multiple tools. We have since added this reference as well as mention of the EnzymeDetector to the discussion (lines 430-444).

***C3:*** *Undeniably, this manuscript presents a wonderful algorithm for genome annotation, which appears to be a significant advance to the state of the art in genome annotation. And if presented strictly on that basis, then this manuscript would be quite suitable and complete, although perhaps not an ideal fit for PLOS Computational Biology. However, the authors present this as a pipeline for automated model reconstruction, which sets the bar much higher. And unfortunately, on that basis, the present manuscript lacks many important details and explanations.*

**A3**: We thank the reviewer for pointing out the absence of these details. We address the questions posed by the reviewer in the following.

***C4:*** *1.) How are biomass reactions predicted? The methods don’t appear to provide details on this. Can the authors supply this information? It seems like this is something that should be in the main text (not just supplement).*

**A4**: Architect performs gap-filling by using user-supplied biomass and currently does not predict biomass reactions. We have clarified this in the text (lines 201-205).

***C5:*** *2.) How do models perform on typical model-based quality measures (e.g. number of gap filled reactions, number of genes in the model, number of blocked reactions)?*

**A5**: These details are found in supplemental table 2. We have also added text to this effect on lines 220-236.

***C6:*** *3.) KEGG in particular contains many reactions that are not suitable for modeling (e.g. reactions involving generic compounds like accepter and donor, strange polymer reactions with incorrect stoichiometry, mixtures of differing levels of stereochemical detail on compounds). Did the authors filter these reactions out? What about charging and proton stoichiometry, which is critical for model accuracy (and typically lacking from KEGG)? Did the authors include this information? What about compartmentation and organelles (KEGG does not specify compartments even in transport reactions)? Do the KEGG based models have transporters?*

**A6:** We acknowledge the difficulties that come with using reactions from the KEGG database. We have performed some level of curation on the original set of reactions we have downloaded from the KEGG database as referred to in the main text (lines 196-198) and detailed in the **Supplemental Text**. Notably, reactions involved in macromolecular processes—identified with a stoichiometry of *n* or the same compound identifier appearing as a substrate and product—were excluded, as well as reactions marked as “incomplete” or “unclear” in KEGG. Following these steps, we further ensured that reactions were balanced by ensuring that the same number of atoms appear on both substrate and product side of reactions. For those reactions we found thus unbalanced, we attempted to balance them individually with typically overlooked compounds like water or protons added to the product or reactant side, and renamed the reaction identifiers with the word “_balanced” appended; reactions that could not be easily balanced were left out in recognizance of the critical need for respecting mass balance. Furthermore, reactions involving generic compound (either identified in KEGG as “generic” or not having a formula) are not considered in either steps of draft reconstruction or gap-filling unless explicitly specified by the user.

Despite our efforts, we recognize that further work may be required to improve the quality of the KEGG-based reaction database used by Architect. For example, we did not consider the differing levels of stereochemical detail on compounds, and did not incorporate information about the charge of each metabolite. Moreover, the KEGG based models do not have defined compartments or organelles. Given that compartment information can be organism-specific, we prefer to provide a metabolic model to the user that they can then further refine for their own purposes (lines 105-108 and lines 428-429). Similarly, transporter information is absent from our version of KEGG. Since prediction of transporter specificity is challenging, for this iteration of Architect, we encourage users to identify transporters separately and include this information when performing metabolic reconstruction. However, as highlighted on lines 466-469, we intend to explore transporter prediction in a future version of Architect, amongst other improvements.

***C7:*** *4.) How do models perform on energetic accuracy? Do they overproduce ATP? Information about how biomass and ATP yields compared to other models would be helpful. Again, this is an incredibly important yet challenging aspect of model reconstruction.*

**A7:** We thank the reviewer for pointing out this important test, which we have since performed. We find that CarveMe models and those made using Architect with BiGG reaction definitions produce biologically realistic ATP yields, unlike Architect models made using KEGG reactions or ModelSEED models (lines 394-417). Thus, we suggest that the level of curation of the reaction database in use has an impact on such simulation results, with BiGG reactions having undergone some level of manual curation as well as further quality assessments for ATP yield when curated by CarveMe’s authors. This further suggests that the KEGG reaction in use by Architect needs further curation, for example, in terms of reaction reversibilities, and that considerations of thermodynamics when gap-filling may also be key (lines 474-477).

***C8:*** *5.) How do models perform on predicting phenotype data? Authors imply essentiality predictions should be improved due to superior annotations, but was this tested?*

**A8**: We have now tested the performance of the *E. coli* model on available Biolog data; in the case of *N. meningitidis*, given the unavailability of such data, we compared the ability of generated models to grow on 6 carbon sources whose ability to sustain growth individually was explored in the creation of the gold-standard model, Nmb_iTM560 (lines 365-392). We find that overall, for *E. coli*, Architect models have similar accuracy to those generated by the other tools, albeit with higher specificity than CarveMe—a possible consequence of the nutrient sources we tested. For *N. meningitidis*, CarveMe models are mostly in agreement with the curated model, followed by Architect-KEGG. Curiously, Architect-BiGG models are constrained to grow on glucose environment only. We suggest that this finding is consistent with Architect’s stringent cutoffs for inclusion of reactions that are not associated with any ECs compared to CarveMe. Additionally, given its complete overlap with reactions in the *E. coli* model, CarveMe’s *N. meningitidis* model may enable growth in conditions irrelevant to the organism.

Additionally, we have tested whether Architect’s annotations produce better essentiality predictions, and we have found this to be the case when the BiGG reaction database was used by Architect (lines 322-363). At the same time, we recognize that all tools have lower recall, and could all benefit from improved gene-protein-reaction annotations.

***C9:*** *6.)What about GPR associations? Do the models include protein complex information in GPRs, and if so, how is this done?*

**A9**: Currently, Architect does not include protein complex information in GPR associations (lines 623-626). Instead, multiple genes associated to a single reaction are linked by an OR relationship. Remarkably, compared to CarveMe and ModelSEED which do predict more complex GPR associations, this shortcoming does not appear to impair Architect-BiGG’s performance on gene essentiality predictions (lines 322-363). However, we recognize (lines 353-363) that capturing complex information in the next iteration of Architect may improve its performance.

***C10:*** *The manuscript appears to contain little detail on these important questions relating to model quality, unless perhaps this information is buried in supplemental material. Of course, much information must always be put in the supplement, but a summary in the main text highlighting that the points above have at least been considered would be most helpful.*

**A10:** We have now included these results in the main text, with additional information in the supplementary.

***C11:*** *It is important to note that a modeling pipeline does not have to be perfect or do all of the things described above to be useful. This pipeline will be valuable on the basis of its improved annotations alone. However, we should be cautious about implying that a pipeline produces higher quality models than competing pipelines without considering the full measure of a model’s quality, which is more than just the annotation accuracy or even MEMOTE score. MEMOTE is wonderful and a great advance as a metric, but it’s not the sole measure of a model’s quality.*

**A11:** We thank the reviewer for their constructive ideas on other ways to measure a model’s quality. Additionally, we acknowledge that additional measures ought to be used to measure a model’s quality in addition to MEMOTE on lines 482-487.

*Reviewer #2: PCOMPBIOL-D-22-00131*

***C12:*** *The manuscript Architect: a tool for producing high-quality metabolic models through improved enzyme annotation by Nursimulu et al. compiles different annotation tools to improve the gene annotation to aid the reconstruction of genome scale metabolic models. Despite some minor wording problems, the manuscript is well-written, and authors do a great job explaining the Architect pipeline. Since the manuscript focuses on annotation, I will recommend changing the name to “Architect: a tool for aiding the reconstruction of high-quality metabolic models through improved enzyme annotation”. The reason of the change is that a tool to produce metabolic models will need to consider:
Addition of constraints to simulate grow under different conditions. Evaluation of accuracy, sensitivity, specificity of predicted growth phenotypes.*

**A12:** We have since evaluated the performance of models produced by Architect in terms of gene essentiality predictions (lines 322-363), growth phenotypes (365-392) and ATP and biomass yield (lines 394-417). However, we acknowledge that Architect produces a draft model from annotation which may require further curation, and have therefore changed the title as suggested by the reviewer.

***C13:*** *Explanation of why authors the selected nematode and two-gram negative bacteria as show case. Does authors tool have a bias?*

**A13:** As we point out in the discussion (lines 445-453), evaluation of metabolic models is complicated by over-representation of model organisms and experimentally sparse information for lesser studied organisms. While focusing on organisms with curated metabolic models, we also sought to achieve a balance through comparisons on a model organism such as *E. coli* and the lesser studied *N. meningitidis*. Furthermore, we chose *C. elegans* to demonstrate the utility of Architect in producing, for a eukaryote, an initial metabolic model with higher-quality annotations.

***C14:*** *In the case of the nematode reconstruction, authors should clarify if their tool has any potential to indicate reaction/enzyme localization at organelle level.*

**A14:** Given that reaction/enzyme localization can be highly organism-specific, Architect does not make any such prediction. We have since added text to the introduction to clarify this point (lines 105-108 and lines 428-429).

***C15:*** *Development of tools for eukaryotic organisms require a comparison with tools such as Plant-SEED.*

**A15**: We have performed the metabolic reconstruction of *C. elegans* using PlantSEED (1), and compare the precision and recall of this model with the other tools as shown in Figure 1 below.

Figure 1: Performance of Architect as a model reconstruction tool for *C. elegans* versus CarveMe, PRIAM, ModelSEED and PlantSEED. Quality of annotations is computed against WormJam over genes found in the curated model, and against UniProt when restricting to those sequences found in the database and with ECs present in the KEGG reaction database. Error bars show the 95% confidence interval for precision and recall, each considered as the estimate of a binomial parameter. P-values, computed using Fisher’s exact test, are calculated only between Architect and other tools individually (with *, ** and *** representing *p* less than 0.05, 0.005 and 0.0005 respectively).

Overall, we note that Architect vastly outperforms PlantSEED both in terms of precision and recall. These results indicate that the scope of the reaction database as well as the training data make a difference in enzyme annotations. In particular, PlantSEED annotations are based on those in *Arabidopsis thaliana* and was built specifically to capture primary metabolism in plant genomes. Therefore, while high-quality kingdom-specific models can be obtained using PlantSEED, its utility is not necessarily transferred to the case of *C. elegans*, whereas Architect is more precise and has higher coverage of metabolic function in this case.

***C16:*** *Architect seems to be a great support tool during the reconstruction process. However, further explanation of supplementary Figure 9 that shows Venn Diagrams comparing Architet-K with CarveMe and PRIAM is needed.*

**A16**: We have now added explanations to the caption of **Supplementary Figure 9** and have included similarly relevant details to the caption of **Supplementary Figure 12**—showing details of the overlap involving Architect-BiGG.

***C17:*** *Authors should discuss the possible source of predicted differences by tool (e.g. example, blast parameters)*

**A17:** We thank the reviewer for this suggestion. We first point to the likelihood score cutoff in Architect which determines which reactions are included in the initial model prior to gap-filling. At each cutoff of 0.1, 0.3, 0.5 (default), 0.7 and 0.9, we reconstructed 20 Architect models with KEGG and BiGG reaction definitions, and calculated precision and recall both in terms of gene-EC annotations and essentiality predictions. We find that variations in this cutoff make little difference overall as we point out in the text (lines 274-281; Supplemental Figure 13 and 14). Additionally, we vary the cutoff used by PRIAM to identify high-confidence reactions in its draft metabolic network, finding predictable decrease in recall and increase in precision with higher (that is, more stringent) cutoffs (Supplemental Figure 15). Overall, it remains that our main findings appear insensitive to the change in cutoff.

***C18:*** *A comparison of Architect with automatic reconstruction tools such as PATRIC is also needed.*	**A18:** We thank the reviewer for this suggestion. Given that PATRIC has been deprecated, we used ModelSEED instead as suggested, and compared its performance on gene-EC annotations (lines 237-273) and on simulation results (lines 322-417).

*Minor*
***C19:*** *Abstract. Is missing some stats of the comparative analysis with other reconstruction tools*

**A19**: We have made this change now (lines 28-30).

***C20:*** *Line 64. PRIAM is not a reconstruction tool*

**A20**: We have clarified the functionality of PRIAM (line 66).

***C21:*** *Line 13. Change constraints by constraint.*

**A21**: This change has been made (line 13).

***C22:*** *Line 37. Rephrase the sentence “-can further …”*

**A22**: This change has been made (line 39).

***C23:*** *Line 40. Change “on a” by “at”*

**A23**: This change has been made (line 42).

References

1.	Seaver SMD, Lerma-Ortiz C, Conrad N, Mikaili A, Sreedasyam A, Hanson AD, et al. PlantSEED enables automated annotation and reconstruction of plant primary metabolism with improved compartmentalization and comparative consistency. Plant J. 2018;95(6):1102-13.
